# Switch to Ritonavir-Boosted versus Unboosted Atazanavir plus Raltegravir Dual-Drug Therapy Leads to Similar Efficacy and Safety Outcomes in Clinical Practice

**DOI:** 10.1371/journal.pone.0164240

**Published:** 2016-10-31

**Authors:** Pierre Gantner, Firouze Bani-Sadr, Rodolphe Garraffo, Pierre-Marie Roger, Michèle Treger, Thomas Jovelin, Pascal Pugliese, David Rey, Dat’AIDS cohort

**Affiliations:** 1 Le Trait d’Union, HIV-infection care center, Nouvel Hôpital Civil, Hôpitaux Universitaires de Strasbourg, Strasbourg, France; 2 Unité des Maladies Infectieuses et Tropicales, Hôpital Robert Debré, Centre Hospitalier Universitaire de Reims, Reims, France; 3 Pharmacologie clinique, Pasteur University Hospital, Nice, France; 4 Infectiologie, Centre Hospitalier Universitaire de Nice, Nice, France; 5 Laboratoire de Biostatistiques, Faculté de Médecine, Strasbourg, France; 6 Service d’Infectiologie, Service des Maladies Infectieuses et Tropicales, Hôtel-Dieu, Centre Hospitalier Universitaire de Nantes, Nantes, France; Universidade do Porto, Faculdade de Farmácia, PORTUGAL

## Abstract

**Objectives:**

To assess immunovirological response, safety and pharmacokinetic of NRTI-sparing regimen dual therapy of atazanavir (ATV) and raltegravir (RAL) in maintenance strategy.

**Methods:**

A retrospective analysis was conducted on a cohort of HIV-infected adults followed in French centers (Dat’AIDS cohort), comparing the proportions of virological and therapeutic failures between ATV + RAL and ATV/ritonavir + RAL dual therapy regimens.

**Results:**

283 patients were assessed: 185 switched for ATV + RAL and 98 for ATV/ritonavir + RAL dual therapy. Virological failure rate at week 96 was 13.8% (95% CI, 9.8–17.8), without difference between the two groups (Log-rank Test, p = 0.87). The cumulative percentages of patients remaining free of therapeutic failure at week 24, 48 and 96 of dual therapy were 74.9% (95% CI, 69.9–80.0), 65.4% (95% CI, 59.8–70.9) and 53.4% (95% CI, 47.5–59.2), respectively. Four out of 39 confirmed virological failures developed RAL resistance. By multivariate analysis, virological failure was associated with high HIV-1 RNA zenith (p = 0.02), low CD4+ T-cell count at baseline (p<0.001) and short duration on antiretroviral therapy (p<0.001). Before week 96, dual therapy was discontinued in 44 patients (16%) because of various adverse events, with no difference between the two groups. Minimal plasma levels were targeted in 84% and 87% of patients for ATV and RAL, respectively, and both were significantly higher in ritonavir-boosted regimen.

**Conclusions:**

Emerging RAL-resistance and discontinuations for adverse events resulted in moderate efficacy rates of ATV and RAL dual therapy in heavily pretreated patients.

## Introduction

HIV-1 antiretroviral therapy (ART) is a life-long treatment. While virological success is achieved in most patients [1], ART often needs to be modified due to adverse events or to prevent long-term toxicity. Although, these long-term complications, have been related to other factors than ART toxicity [2,3] such as a higher prevalence of traditional risk factors [4,5] and HIV replication [6], preventing drug-related toxicity should be considered for ART switch.

To decrease drug burden, reducing from standard ART to a two or one drug-containing regimen has been evaluated, with conflicting results on virological efficacy [7,8]. Indeed, switching to maraviroc and raltegravir dual therapy was too “weak” to maintain virological success [7], with a high risk of emerging resistance. Besides, no predictors of virological failure were identified [9]. Conversely, darunavir/ritonavir or lopinavir/ritonavir monotherapy exhibited efficacy rate concordant with triple drug regimen [10,11], with no selection of major resistant variants at failure [12]. However, due to insufficient diffusion in cerebro-spinal fluid of the boosted protease inhibitor, a sanctuary replication in central nervous system was noticed [13].

In these studies, a shorter time on ART before monotherapy, which was consistent with a high HIV reservoir, was predictive of virological failure [14]. Yet, NRTI-sparing regimens remain an interesting but under-evaluated option for drug-reducing strategies. Dual therapy including raltegravir (RAL), an integrase inhibitor, and atazanavir, a protease inhibitor, (with or without ritonavir booster, ATV or ATV/r) is such an option. A single randomized trial with naïve patients starting ATV and RAL dual therapy was discontinued because of high rates of emerging RAL resistance [15], whereas pilot studies in maintenance strategies showed short-term efficacy [16–19]. These studies were conducted on small groups of patients, mostly evaluating ritonavir-boosted strategies, with no assessment of lipid profiles. Besides, some of these studies reported grade 3 or 4 total bilirubin abnormalities [15,16] that were maybe associated with the ATV dosing strategies.

Besides, RAL or ATV-containing regimen may have a favorable impact on immune activation [20–25]. Although controversial, it seems that both RAL and ATV (especially unboosted)-based regimen show improvements in lipid profiles [26–29]. Thus, switching to a combination including RAL and ATV in ART-experienced patients is an ongoing studied option [30].

In the present study, we aimed to assess viral suppression of ART-experienced patients switching to dual therapy on a large cohort of patients, allowing comparison between boosted and unboosted-ATV regimen. As secondary outcomes, we assessed safety, drug plasma trough concentrations and predictors of virological failures and dual therapy discontinuations.

## Methods

### Study design

The Dat’AIDS cohort represents a collaboration of major French HIV treatment centers. These centers maintain prospective cohorts of all HIV-1-infected patients who provided written consent. The cohorts are implemented via a common electronic medical record [31]. Past and prospective clinical events, laboratory tests and therapeutic history are routinely gathered with appropriate dates. All participants gave prospective written consent for their clinical data to be anonymized and then analyzed for research purposes (Commission Nationale de l’Informatique et des Libertés). The local Ethics Committee of the Medicine Faculty, Strasbourg, France, approved the study.

We performed a retrospective longitudinal analysis on HIV-1-positive adults participating to the Dat’AIDS cohort who were ART-experienced and were switched to ATV+RAL or ATV/r+RAL regimen between January 2008 and June 2014, whatever baseline CD4+ T-cell count or HIV-1 RNA. Comparing the efficacy, measured by the proportion of therapeutic and virological success at week 24, 48 and 96, between the two regimens was assessed as the primary outcome measure. Confirmed virological failure was defined as a plasma HIV-1 RNA > 50 copies/mL based on two consecutive measurements within a month or HIV-1 RNA > 1000 copies/mL on one sample. Therapeutic failure was defined by dual therapy discontinuation whatever the reason or confirmed virological failure. Secondary objectives were predictors of virological and therapeutic failure, safety and drug plasma levels assessment through follow-up.

### Data collection

Demographic data, HIV disease status, previous ART, reason for changing therapy, steady-state plasma levels of RAL and ATV, laboratory parameters; in particular plasma HIV-1 RNA, CD4+ and CD8+ T-cell count, total bilirubin, alanine aminotransferase (ALT), aspartate aminotransferase (AST), serum creatine phosphokinase (CPK), lipids, creatinine and estimated glomerular filtration rate (eGFR) was obtained using the MDRD formula were extracted from recorded medical charts. Laboratory abnormalities were graded using the 2003 ANRS scale (http://www.anrs.fr). Genotypic resistance testing was performed at virological failure and was interpreted according to the 2015 ANRS algorithm (http://www.hivfrenchresistance.org) according to the current French guidelines [32].

### Statistical considerations

Dual therapy efficacy was analyzed according to the time to loss of therapeutic and virological response (TLOVR) algorithm. The TLOVR was estimated using the Kaplan-Meier method.

The Student’s *t*-test was used to compare any changes in quantitative variables. Categorical variables were compared using the χ^2^ test or Fisher’s exact. All tests were two-tailed and performed with a level of statistical significance of 0.05. Between-strategy mean difference in trough concentrations was evaluated by calculating the geometric mean ratio (GMR) with a 90% confidence interval (CI).

Predictors of *virological and therapeutic failure*, *with a special attention to therapeutic failure for adverse events* were assessed. Factors considered were plasma viral load (plasma HIV RNA < 50 HIV-1 RNA copies/mL versus > 50 copies/mL), history of virological failure (yes and no), age, gender, risk behaviour for HIV transmission, CDC stage, current CD4 count, CD4 nadir, viral load zenith, delay since HIV infection diagnosis, duration with HIV-1 RNA <50 copies/mL on ART status, previous ART regimen, reason for switching and boosted or not dual therapy. Predictors of *virological and therapeutic failure* were also assessed in a focus on patients with HIV-1 RNA <50 copies/mL at baseline. The differences between groups in characteristics at baseline were evaluated using non-parametric tests (Wilcoxon and Fisher’s exact tests). Variables achieving a *p* value <0.17 were entered into a multivariate logistic regression model with a backward stepwise method based on the likelihood ratio test. Adjusted odd-ratios (aOR) are given with a 95% CI. Statistical analyses were performed with R software (R Foundation, Vienna, Austria).

Data are presented as number (percentage) or median (interquartile range 25–75% [IQR]).

## Results

### Participant Disposition

A total of 283 patients were analyzed: 185 switched for ATV+RAL and 98 switched ATV/r+RAL dual therapy; characteristics are depicted in Table 1. Patients were followed for a median of 95 weeks (IQR, 24–185). One hundred twenty-seven patients (45%) had past history of confirmed virological failure. At baseline, 80% of patients had HIV-1 RNA <50 copies/mL and the remaining 20% had a median plasma HIV-1 RNA of 663 copies/mL (IQR, 159–37095), median CD4+ T-cell count was 552/mm^3^ (IQR, 391–717). Before switching to dual therapy, 56% and 20% of patients were already on an ATV and RAL-based regimen, respectively. The main reason of switching to dual therapy was for ART-related adverse events (47%). On dual therapy, patients received primarily ATV 200 mg BID (42%) and 400 mg QD (21%).

**Table 1:** Patients’ characteristics at baseline.

*Characteristic*	*Overall* (n = 283)	*ATV+RAL* (n = 185)	*ATV/r+RAL* (n = 98)	*p value*
Male gender, n (%)	173 (61%)	106 (57%)	67 (68%)	0.07
Age, years, median (IQR)	53 (48–62)	52 (47–60)	56.5 (49–66)	0.01
Time since HIV diagnosis, years, median (IQR)	16 (10–20)	15 (9–19)	17 (12–20)	0.03
Time on antiretroviral therapy, years, median (IQR)	12 (7–15)	12 (7–14)	13 (9–15)	0.03
History of AIDS defining events, n (%)	91 (32%)	54 (29%)	37 (38%)	0.1
Co-infection with HCV, n (%)	30 (11%)	18 (10%)	12 (12%)	0.5
Co-infection with HBV, n (%)	5 (2%)	4 (2%)	1 (1%)	0.7
BaselineHIV-1 RNA <50 copies/mL, n (%)	227 (80%)	150 (81%)	77 (79%)	0.6
Time with HIV-1 RNA <50 copies/mL, years, median (IQR)	9 (7–14)	9 (5–14)	10 (8–13)	0.1
Zenith HIV-1 RNA, log copies/mL, median (IQR)	4.98 (4.45–5.48)	4.88 (4.31–5.45)	5.13 (4.58–5.59)	0.04
Baseline CD4+ T-cell count, cells/mm^3^, median (IQR)	552 (391–717)	527 (388–713)	593 (398–726)	0.3
Nadir CD4+ T-cell count, cells/mm^3^, median (IQR)	172 (72–254)	191 (72–262)	149 (54–243)	0.1
Baseline CD4+/CD8+ T-cell ratio, median (IQR)	0.63 (0.42–0.82)	0.64 (0.42–0.83)	0.62 (0.42–0.81)	0.5
Total bilirubin, μmol/L, median (IQR)	16 (9–34)	16 (8–32)	20 (9–36)	0.8
ALT, IU/L, median (IQR)	26 (18–42)	22 (18–37)	29 (20–52)	0.04
AST, IU/L, median (IQR)	28 (22–39)	26 (21–34)	32 (24–47)	0.002
CK, IU/L, median (IQR)	110 (78–219)	105 (78–188)	150 (81–234)	0.06
Creatinine, μmol/L, median (IQR)	76 (64–99)	74 (61–97)	80 (69–99)	0.6
eGFR, mL/min, median (IQR)	83 (64–102)	83 (62–103)	83 (65–99)	0.8
Lipid profile, mmol/L, median (IQR)				
Total cholesterol	5.33 (4.40–6.16)	5.24 (4.18–6.10)	5.43 (4.63–6.18)	0.5
HDL	2.96 (2.05–3.60)	2.96 (2.19–3.75)	2.98 (2.01–3.52)	0.5
LDL	1.16 (0.78–1.52)	1.16 (0.75–1.50)	1.17 (0.89–1.68)	0.2
Triglycerides	1.69 (1.10–1.55)	1.68 (1.10–2.57)	1.69 (1.17–2.53)	0.8
Previous antiretroviral regimen, n (%)				0.1
2 NRTI + 1 PI±r	128 (45%)	75 (41%)	53 (54%)	
2 PI±r	28 (10%)	15 (8%)	13 (13%)	
2 NRTI + 1 NNRTI	24 (9%)	18 (10%)	6 (6%)	
1 PI±r + 1 INI	24 (9%)	19 (10%)	5 (5%)	
2 NRTI + 1 INI	21 (7%)	15 (8%)	6 (6%)	
1 NRTI + 1 PI±r	12 (4%)	6 (3%)	6 (6%)	
3 NRTI	11 (4%)	10 (5%)	1 (1%)	
Others	35 (12%)	27 (15%)	8 (8%)	
Number of previous antiretroviral regimen, median (IQR)	7 (4–11)	6 (4–11)	8 (5–11)	0.02
Reasons for switching to dual therapy, n (%)				0.2
Adverse events	134 (47%)	80 (43%)	54 (55%)	
End of treatment/protocol	31 (11%)	26 (14%)	5 (5%)	
Virological failure	27 (10%)	17 (9%)	10 (10%)	
Preventing toxicity	17 (6%)	13 (7%)	4 (4%)	
Simplification	15 (5%)	9 (5%)	6 (6%)	
Patients’ personal decision	11 (4%)	8 (4%)	3 (3%)	
Pharmacological adjustment	10 (4%)	5 (3%)	5 (5%)	
Other reasons	38 (13%)	27 (15%)	11 (11%)	

ATV or ATV/r: Atazanavir or Ritonavir-boosted Atazanavir; HDL: High-density lipoprotein; IQR: Interquartile Range; LDL: Low-density lipoprotein; NNRTI: Non-Nucleoside Reverse Transcriptase Inhibitor; NRTI: Nucleoside Reverse Transcriptase Inhibitor; PI±r: Protease Inhibitor ± ritonavir; RAL: Raltegravir.

### Virological response

**Fig 1:**
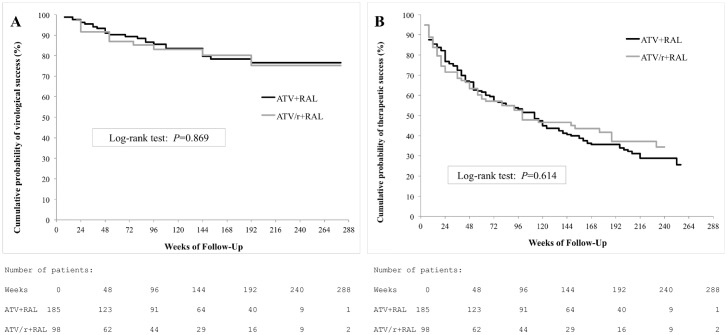
Cumulative probability of treatment success according with ritonavir association. Survival curves by time to loss of virological response analysis, according to virological failure (A) and treatment failure (B). ATV/r or ATV: Boosted or Unboosted Atazanavir, RAL: Raltegravir.

The cumulative percentages of patients remaining free of confirmed virological failure on dual therapy were 93.3% (95% CI, 90.4–96.2) at week 24, 89,4% (95% CI, 85.8–93) at week 48 and 86.2% (95% CI, 82.2–90.2) at week 96, without difference between ritonavir-boosted and unboosted strategies (Fig 1A). Before week 96, confirmed virological failure occurred in 39 patients (14%) [26 (14%) in the ATV+RAL group; 13 (13%) in the ATV/r+RAL group] with HIV-1 RNA ranging from 62 to 100000 copies/mL. In the multivariate analysis (Table 2), the risk of virological failure was strongly associated with a high historical HIV-1 RNA zenith (aOR per log copies/mL, 1.74 [1.02–2.98]; p = 0.02), a low CD4+ T-cell count at baseline (aOR per 100/mm^3^, 0.72 [0.65–0.89]; p<0.001) and a short duration on ART (aOR per year since ART initiation, 0.90 [0.84–0.96]; p<0.001). A past history of virological failure and a detectable HIV-1 RNA at baseline were not associated with an increased risk of virological failure on dual therapy. When focusing on patients with HIV-1 RNA < 50 copies/mL at baseline (n = 227), predictors of virological failure were similar to the entire cohort.

**Table 2:** Significant predictors associated with virological failure and therapeutic failure.

	Virological failure (n = 283)		Univariate analysis (p)	Multivariate analysis (p)	OR (95% CI)
	Yes (n = 39)	No (n = 244)			
Median historical HIV-1 RNA zenith, log copies/mL (IQR)	5.2 (5.0–5.7)	4.9 (4.3–5.4)	<0.001	0.02	1.74^a^ (1.02–2.98)
Median CD4+ T-cell count, /mm^3^ (IQR)	429 (241–571)	572 (402–728)	0.002	<0.001	0.72^b^ (0.65–0.89)
Median duration on ART, year (IQR)	10 (4–13)	12 (8–15)	0.01	<0.001	0.90^c^ (0.84–0.96)
	Therapeutic failure (n = 283)		Univariate analysis (p)	Multivariate analysis (p)	OR (95% CI)
	Yes (n = 132)	No (n = 151)			
Median CD4+/CD8+ T-cell ratio (IQR)	0.6 (0.4–0.8)	0.7 (0.5–0.9)	0.02	0.02	0.38^d^ (0.14–0.97)
C CDC stage, n (%)	51 (39%)	40 (26%)	0.02	0.05	1.84 (0.99–3.45)
Median duration on ART, year (IQR)	12 (7–14)	12 (8–15)	0.04	0.03	0.95^c^ (0.90–0.99)

CI, Confidence Interval; IQR, Interquartile Range; OR, Odd-Ratio.

^a^, per log copies/mL;

^b^, per 100/mm^3^;

^c^, per year;

^d^, per ratio unit.

Both past and at failure genotypic resistance testing was available in 31/39 participants with confirmed virological failure, no resistance to RAL and ATV were detected before switching. Four out of 31 confirmed virological failures with available genotypes developed RAL resistance: two of them had emerging Y143C (week 24 and week 48), one patient had N155H (week 48) and the last patient had Q148R (week 48). Neither ATV nor other RAL resistance mutations were observed.

### Therapeutic response

The cumulative percentages of patients remaining free of therapeutic failure at week 24, 48 and 96 of dual therapy were 74.9% (95% CI, 69.9–80.0), 65.4% (95% CI, 59.8–70.9) and 53.4% (95% CI, 47.5–59.2), respectively (Fig 1B). By multivariate analysis (Table 2), therapeutic failure was associated with a low CD4+/CD8+ T-cell ratio at baseline (aOR per ratio unit, 0.38 [0.14–0.97]; p = 0.02), C CDC stage (aOR, 1.84 [0.99–3.45]; p = 0.05) and a short duration on ART (aOR per year since ART initiation, 0.95 [0.90–0.99]; p = 0.03). There was no difference between ritonavir-boosted and unboosted strategies. Before week 96, 132 (46.6%) patients stopped dual therapy (Table 3), partly because of various adverse events attributed to the new regimen (n = 44). These were renal toxicity (n = 8), gastrointestinal disturbances (n = 5), cutaneous effects (n = 5), hyperbilirubinemia (n = 4), neuropsychiatric side effects (n = 4), dyslipidemia (n = 3), liver toxicity (n = 3), mitochondrial toxicity (n = 3) and others (n = 9). The multivariate analysis identified that a high number of previous ART regimen was a significant predictor of stopping dual therapy due to adverse event (aOR per previous ART regimen, 1.07 [1.00–1.14]; p = 0.03).

**Table 3:** Reasons for stopping dual therapy before week 96 (n = 132).

	*Overall* (n = 132)	*ATV+RAL* (n = 86)	*ATV/r+RAL* (n = 46)	*p value*
Reasons for stopping dual therapy, n (%)				0.522
Adverse events	44 (33%)	24 (28%)	20 (44%)	
Virological failure	39 (30%)	26 (30%)	13 (28%)	
With emerging RAL resistance	4	3	1	
With emerging ATV resistance	0	0	0	
Patients’ personal decision	13 (10%)	10 (12%)	3 (7%)	
Pharmacological adjustment	6 (5%)	4 (5%)	2 (4%)	
Pregnancy project or current	3 (2%)	2 (2%)	1 (2%)	
Other reasons	27 (20%)	20 (23%)	7 (15%)	

ATV or ATV/r: Atazanavir or Ritonavir-boosted Atazanavir; RAL: Raltegravir.

### Laboratory outcomes

The CD4+ T-cell count increased, with a mean increase at week 96 of 60 cells/mm^3^ (95% CI, 19–100, p = 0.005). Similarly, the CD4+/CD8+ T-cell increased on study, with a mean increase at week 96 of 0.21 (95% CI, 0.08–0.35, p = 0.003).

**Fig 2:**
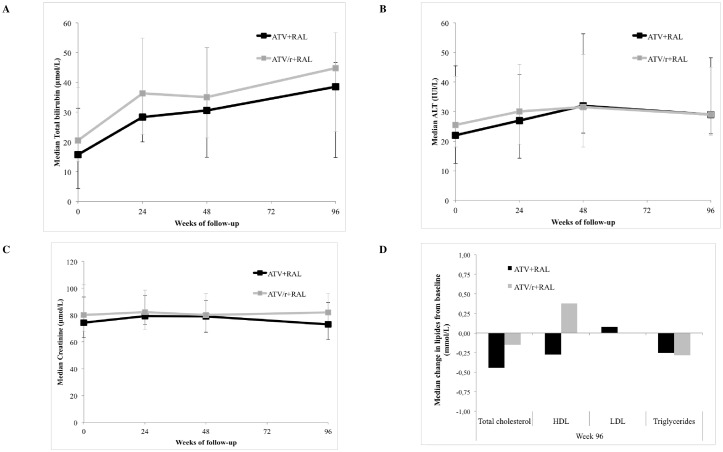
Changes from baseline in main laboratory parameters. ATV/r or ATV: Boosted or Unboosted Atazanavir; RAL: Raletgravir; ALT: Alanine aminotransferase; HDL: High-density lipoprotein; LDL: Low-density lipoprotein.

Only mild to moderate laboratory abnormalities were found on follow-up. There was a significant elevation in total bilirubin levels, with a mean increase of 17 μmol/L at week 24 (95% CI, 10–23, p<0.0001), with no difference between the two groups (Fig 2). However, there was no significant change in ALT, AST, CK, creatinine and eGFR during follow-up. In the ATV/r+RAL group, there were no significant differences in total cholesterol, high-density lipoprotein (HDL), low-density lipoprotein (LDL) cholesterol or triglyceride levels at different time points of follow-up. However, in the ATV+RAL group, there were significant reductions from baseline in: total cholesterol level with a mean reduction of 0.82 mmol/L (95% CI, 0.19–1.45, p = 0.01), HDL level with a mean reduction of 0.40 mmol/L (95% CI, 0.04–0.75, p = 0.03), and triglyceride level with a mean reduction of 0.49 mmol/L (95% CI, 0.03–0.97, p = 0.04) at 96 weeks of treatment.

### Pharmacokinetics

A total of 155 and 135 ATV and RAL plasma measurements were assessed, respectively. Overall, median ATV and RAL concentrations were of 550 ng/mL (IQR, 300–1195) and 121 ng/mL (IQR, 55–289), respectively. During follow-up, targeted minimum trough ATV concentrations (200 ng/mL) were achieved in 84% of patients and 87% of patients had a RAL level above the 95% inhibitory concentration (15 ng/mL) [32].

The geometric mean trough concentrations of both ATV and RAL are shown in Table 4. Once daily RAL was associated with lower steady-state values than twice daily, with a GMR of 0.40 (90% CI, 0.22–0.72). Both ATV and RAL concentrations were significantly higher in ritonavir-boosted regimen, with a GMR of 1.81 (90% CI, 1.19–2.78) and 2.04 (90% CI, 1.25–3.34), respectively. Unboosted ATV concentrations were similar between 400 mg QD and 200 mg BID-dosing strategies.

**Table 4:** ATV and RAL pharmacokinetic parameters in different dosing strategies.

	Dosage, n	Dosage Timing	C_trough_ Geometric mean, ng/mL (90% CI)	GMR (90% CI)
ATV	155			
With RTV	40	NA	766.5 (506.5–1160.0)	1.81 (1.19–2.78)
Without RTV	115	NA	424.5 (345.4–521.6)	
200 mg BID (without RTV)	53	12 h	337.1 (237.9–477.6)	1.08 (0.50–2.36)
400 mg QD (without RTV)	16	24 h	311.8 (123.9–784.8)	
300 mg BID (without RTV)	22	12 h	866.8 (689.0–1090.6)	2.35 (1.64–3.35)
600 mg QD (without RTV)	11	24 h	369.4 (280.9–485.6)	
RAL	135			
With RTV	92	NA	141.0 (107.3–185.1)	2.04 (1.25–3.34)
Without RTV	43	NA	69.1 (44.6–107.1)	
800 mg QD	24	24 h	52.7 (29.5–94.3)	0.40 (0.22–0.72)
400 mg BID	111	12 h	132.3 (102.8–170.2)	

ATV: Atazanavir; CI, Confidence interval; Ctrough: Trough Concentration; GMR: Geometric Mean Ratio; NA: Not applicable; RAL: Raltegravir; RTV: Ritonavir.

Both ATV and RAL high trough concentrations were not linked to therapeutic failure or discontinuations due to adverse events.

## Discussion

Long-life ART poses significant challenge to effective and tolerated therapeutic intervention, especially among heavily pretreated and ageing patients. Indeed, treatment modification is not always simple in heavily ART-experienced patients, due to previous adverse events and/or ART resistance mutations. Evaluating a NRTI-sparing regimen in a large prospective cohort, we showed that ATV and RAL dual therapy had moderate efficacy for lengthy period of time in these patients, mostly due to adverse events and virological failures. Moreover, an emerging RAL-resistance risk at virological failure was encountered (0.4% at week 24, 1.4% at week 48 and 1.4% at week 96). We showed that there was no significant difference between ritonavir-boosted and unboosted strategies in efficacy and tolerance laboratory parameters, except for lipid profiles.

Other studies showed moderate efficacy rates of this dual therapy [16–19,30]. The HARNESS study enrolled experienced patients, with undetectable viremia, randomized to receive ATV/r+RAL or ATV/r plus tenofovir/emtricitabine [30]. At week 48, switching to ATV/r+RAL resulted in lower therapeutic success rate (69.4%) than ATV/r plus tenofovir/emtricitabine (86.5%). In our real-life study, we found similar rates of virological suppression at week 48 (65.4%) compared to the HARNESS trial. Similarly to the SPARTAN study, RAL-resistance emerged among patients experiencing virological failure (4/31 patients with available genotypes) [15]. Rates of emerging resistance in subjects who failed dual therapy further support resistance testing in case of virological failure. Although we did not perform a multiple comparison adjustment, we identified significant predictors of virological failure on dual therapy by multivariate analysis. Virological failure on dual therapy was strongly associated with high HIV-1 RNA zenith and low CD4+ T-cell count at baseline, whether patients had HIV-1 RNA <50 or >50 copies/mL at baseline. Both parameters have been correlated with high HIV reservoir levels [33,34]. So, we might hypothesize there is a link between HIV reservoir and virological failure on dual therapy. Measuring HIV-1 DNA before switching might help adapting simplification strategies and select patients [14,35]. Besides, a short duration on ART has also been associated with virological failure. This might be related with time spent on suppressed plasma HIV-1 RNA under successful ART before switching to dual therapy. Indeed, long-term virological control was associated with decreased rates of low-level viremia [36] and HIV reservoir [37]. We can hypothesize that this two drug-containing regimen was not efficient enough to maintain viral suppression among patients who recently obtained virological control.

Regarding laboratory abnormalities, dual therapy showed moderate toxicity rates as 44 (16%) participants discontinued because of adverse events through week 96. Lower rates of discontinuations over a similar period and for a similar patient population have been reported with other regimen switches [8]. Higher rates of discontinuations due to adverse events were found for highly ART-exposed patients. Results of bilirubin elevations presented herein are similar if not actually better than those of the pilot studies where 13.0 to 20.6% of grade 3 or 4 total bilirubin abnormalities were described [15,16,30]. Switching for ATV+RAL yielded a significant decrease in triglycerides, HDL and total cholesterol between baseline and week 96. This result is surprising as some other ART regimen showed lipid-lowering effects, such as tenofovir [38]. In our study, this may be due to ritonavir stopping when switching to dual therapy [39].

Pharmacokinetic results supported the twice-daily use of RAL 400 mg and were in keeping with data from the literature [17,40,41]. Coadministration of ATV and RAL is known to increase RAL exposures [42–44], which explain why both ATV and RAL trough concentrations increased with ritonavir association. Although, ATV exposure was higher with ritonavir-boosted regimen, bilirubin elevation was not significantly different from unboosted regimen. Of major importance, a high proportion of patients achieved targeted trough concentrations for both ATV and RAL on dual therapy.

To the best of our knowledge, this is the first study that compared long-term immunovirological outcomes of switching to ATV and RAL dual therapy between boosted and unboosted strategies. Although this study has several limitations inherent to the DAT’AIDS cohort design, as the retrospective design and the mixed population (patients with both undetectable and detectable viremia at baseline) [31], we confirmed a moderate efficacy and safety profile of switching to ATV and RAL dual therapy in a “real-life” setting [30]. All efficacy and safety parameters were similar in patients treated with ATV+RAL versus ATV/r+RAL. Our results suggest that dual therapy could possibly be initiated in selected patients, according to the risk of virological failure that may be linked to a high HIV reservoir, and the risk of emerging RAL-resistance at virological failure. Patients with favorable immunovirologic profile (i.e. low HIV reservoir with a long duration on efficient ART) might benefit from drug reducing to dual ATV and RAL therapy.
